# Your Spreadsheets Can Be FAIR: A Tool and FAIRification Workflow for the eNanoMapper Database

**DOI:** 10.3390/nano10101908

**Published:** 2020-09-24

**Authors:** Nikolay Kochev, Nina Jeliazkova, Vesselina Paskaleva, Gergana Tancheva, Luchesar Iliev, Peter Ritchie, Vedrin Jeliazkov

**Affiliations:** 1 Department of Analytical Chemistry and Computer Chemistry, Faculty of Chemistry, University of Plovdiv, 24 Tsar Assen St, 4000 Plovdiv, Bulgaria; vessy@uni-plovdiv.net (V.P.); gerganatancheva1@gmail.com (G.T.); 2 Ideaconsult Ltd., 4 Angel Kanchev St, 1000 Sofia, Bulgaria; luchesar.iliev@gmail.com (L.I.); vedrin.jeliazkov@gmail.com (V.J.); 3 Institute of Occupational Medicine, Research Avenue North, Riccarton, Edinburgh EH14 4AP, UK; peter.ritchie@iom-world.org

**Keywords:** nanoparticle, Excel spreadsheet, eNanoMapper database, JSON, software parser, nanosafety, data, metadata, FAIR data

## Abstract

The field of nanoinformatics is rapidly developing and provides data driven solutions in the area of nanomaterials (NM) safety. Safe by Design approaches are encouraged and promoted through regulatory initiatives and multiple scientific projects. Experimental data is at the core of nanoinformatics processing workflows for risk assessment. The nanosafety data is predominantly recorded in Excel spreadsheet files. Although the spreadsheets are quite convenient for the experimentalists, they also pose great challenges for the consequent processing into databases due to variability of the templates used, specific details provided by each laboratory and the need for proper metadata documentation and formatting. In this paper, we present a workflow to facilitate the conversion of spreadsheets into a FAIR (Findable, Accessible, Interoperable, and Reusable) database, with the pivotal aid of the NMDataParser tool, developed to streamline the mapping of the original file layout into the eNanoMapper semantic data model. The NMDataParser is an open source Java library and application, making use of a JSON configuration to define the mapping. We describe the JSON configuration syntax and the approaches applied for parsing different spreadsheet layouts used by the nanosafety community. Examples of using the NMDataParser tool in nanoinformatics workflows are given. Challenging cases are discussed and appropriate solutions are proposed.

## 1. Introduction

The nanotechnology field is an increasingly dynamic area in materials science research and development, introducing novel materials with unique properties due to their size in the range of nanometers [1]. Nowadays nanoparticles and nanostructured materials represent an active area of research [1] motivated by interesting properties of manufactured nanomaterials (NMs), which significantly improve the characteristics of bulk materials, in terms of strength, conductivity, durability, lightness, and many other useful properties. Nanomaterials are used across almost all industrial sectors [2] where the “beautiful” properties of nanomaterials inspire widespread usage, but also raise questions and concerns about their influence on human health and on the environment [3]. Specific nanomaterial properties, like surface area, influence various bio interactions and health effects such as: entry to the blood stream; translocation in cells, tissues, and functional organelles; raising the risk of toxic effects, such as inflammation, genotoxicity, autophagy, neurotoxicity, and ultimately cell death [4]. All safety issues regarding nanomaterials and nanotechnology—as evaluated by toxicology, ecotoxicology, exposure assessment, mechanism of interaction, risk assessment and standardization—fall into the domain of nanosafety, and the need for efficient and Findable, Accessible, Interoperable, and Reusable (FAIR) nanosafety data is well recognized in this domain.

Over the last decade, the field of nanoinformatics has rapidly developed to provide data driven solutions in the area of nanomaterials safety [5]. Safe by Design approaches are encouraged and promoted through regulatory initiatives and multiple scientific projects. Experimental data are at the core of nanoinformatics processing workflows for risk assessment. Database technologies are extensively used for storing NM information, enabling query and analysis of chemical and physical properties, bio assay experiments, and impacts on humans and nature, especially in the context of nanosafety and risk assessment.

The efficient aggregation of NM data from multiple sources is possible only if the original measurements are coupled with rich metadata. Metadata management is at the core of FAIR principles, while the efforts of the experimentalist are mostly focused on experimental data generation. The FAIR principles—Findable, Accessible, Interoperable, and Reusable (meta)data—allow experimental data to be used beyond its origin, for solving scientific problems, data gap filling, reading across applications, material and property modelling and supplying tools for other needs of science, industry, and regulators. The ubiquitous use of non-FAIR data entry formats (e.g., Excel spreadsheets) can be a stumbling block towards FAIR data, thus requiring steps to ensure FAIR principle compliance—a FAIRification process [6]. A generic FAIRification workflow includes several steps: gaining access to the data, analysis of the data and metadata, defining a semantic model for the data and metadata, making data and metadata linkable, and finally, deployment (hosting the FAIR data and providing human- and machine-readable access).

The underlying semantic data model is crucial to the efficiency of any nanoinformatics workflow, thus the NM representation in chemical databases is a primary subject of the new and rapidly evolving field of nanoinformatics. The substance definition in the European Union regulation REACH [7] and in the Classification, Labelling and Packaging (CLP) Regulation is applicable to all forms of substances and materials on the market, including nanomaterials. This aligns with our practical approach for treating NM data as a particular case of chemical substances. We have previously reviewed several major data model approaches for representing NM information [8,9]. IUCLID, the recommended software for submission of dossiers by ECHA [10], stores and maintains summary data on the hazardous properties of chemical substances and mixtures, as well as their use and associated exposure levels [11]. IUCLID’s data model is the reference implementation of the OECD harmonized templates (OHT) [12]. The BioAssay Ontology (BAO) provides a foundation for standardizing assay descriptions and endpoints; high-throughput screening (HTS) data are represented using expressive description logic [13] with the main objectives of enabling effective integration, aggregation, retrieval, and analyses of drug screening data [14]. The CODATA/Vamas Uniform Description System (UDS) [15] supports the principle that data produced by research and susceptible to use for further research should be as open as possible and as closed as necessary. The UDS 2.0 framework [16] identifies some basic information categories: General Identifiers, Characterization, Production, Specification used to describe nanomaterials, as well as subcategories and descriptors giving detailed data.

The ISA data model defines three basic layers for sharing metadata related to experiments: Investigation, Study, and Assay, as well as the actual experimental data stored on a separate fourth layer [17,18]. The ISA model can be serialized via different formats, such as ISA–Tab [18] (stored in spreadsheet files) or ISA–JSON [19] (serialized in the more convenient JSON format).

The eNanoMapper database is an open source chemical substance data management solution [8], which currently possesses the largest searchable compilation of nanoEHS (Nano Environment, Health and Safety) data in Europe (https://search.data.enanomapper.net/), from multiple completed and most of the current H2020 Nano–EHS projects. Large numbers of nanosafety data sets, generated by past NanoSafety Cluster (NSC) projects have been integrated (NANoREG, NanoTest, MARINA, ENPRA, NanoGenotox, caLIBRAte, NanoReg2) using a data model, which is also successfully applied to manage chemical substances and safety data from ECHA dossiers. The eNanoMapper data model is inspired by the data models described above—OECD Harmonized Templates (OECD HT), CODATA, BioAssay ontology and ISA–Tab—and was previously described in [8].

Regardless of the existing structured data representations and formats, in practice the nanosafety data is predominantly initially recorded in custom spreadsheet templates by the experimental scientists. Although spreadsheet files are convenient for the experimentalists to use, they also pose great challenges during their subsequent import into databases, due to variability in data representation (inconsistent cell formats, different layouts and metadata specific to each laboratory, missing or insufficient metadata, etc.). In fact, the most resource intensive and time consuming process of any FAIRification process [6] is not the terminology alignment, but defining the semantic model, or, in the case of an existing data model, the alignment between the semantic models of the input data formats and the data storage.

In this paper, we present a tried and tested FAIRification workflow, developed for the eNanoMapper database during the data integration activities across several nanosafety projects. The data curation process includes the mapping of the (implicit) semantic model of the Excel spreadsheets onto the eNanoMapper data model. The central player is NMDataParser—an open source configurable Excel spreadsheet parser, enabling data curators to map particular Excel file layouts onto the data model of the eNanoMapper database. The parser configuration (the map) is defined by additional JSON files. We describe the JSON configuration syntax of these files for parsing selected spreadsheet layouts used by the nanosafety community. The NMDataParser tool can be used as a component of a data import workflow or as a standalone application to convert data from non-FAIR Excel files into different structured semantic formats (RDF, JSON–LD, JSON, ISA–JSON). Examples are given for using NMDataParser in various nanoinformatics workflows. Challenging cases are discussed and solutions are proposed.

## 2. Materials and Methods

### 2.1. Data Entry Templates

There are several existing approaches to support data entry of experimental results for regulatory purposes (e.g., OHTs), research data in bioinformatics (e.g., ISA–Tab, ISA–JSON) and its extensions for NM (e.g., ISA–Tab–Nano), as well as data logging templates originating and widely used in EU funded projects, summarized in the NanoInformatics Roadmap 2030, Chapter 5 [5]. While any data management system will prefer a structured format for data import, the data providers are much more likely to adopt a data entry centric workflow that requires the use of familiar tools and a format that follows the design of the experiment and is not too rigid. The lesson learned during the last decade is that unless there is external pressure to use a particular tool or structured format, the data generators resort to Excel spreadsheets, given their ubiquity and convenience. Thus, the approach taken early on by eNanoMapper was to develop a tool to help in converting the existing variety of spreadsheets into a common semantic data model, incorporate annotation with ontology terms, and steer the spreadsheet layout design towards harmonization, while retaining the user friendliness and features deemed important by the labs. We have been successful with parsing and importing into the eNanoMapper databases more than 1500 Excel spreadsheet files. These files roughly follow the NANoREG/JRC and IOM types of templates (briefly described below), and the subsequent sections provide specific layout examples.

The NANoREG approach to the harmonisation of data logging is based on structuring the information on assays in a set of MS Excel templates, developed by JRC and released under the Creative Commons Share-Alike license [20]. While not strictly following the ISA–Tab and ISA–Tab–Nano specifications, the templates have been designed around “ISA–Tab logic”, i.e., structuring the data in investigation–study–assay-related groups. The motivation to create new templates instead of using ISA–Tab/ISA–Tab–Nano ones is the perceived low applicability of the latter in a more “lab-related” data logging due to lack of user-friendliness. The NANoREG/JRC templates are being used for data entry in a number of EU funded projects and a subset of the physicochemical templates was refined and published by the H2020 GRACIOUS project [21]. A high level overview follows:

1. The sample information group describes the NM (including names, ID, supplier, vial number and replicate number, as well as dispersant). The reporting organisation, operator, and date of the experiment are also in this section;
2. An unnamed group listing the module (physicochemical, in-vitro, or in-vivo), the endpoint (e.g., cell viability), and the assay name (e.g., “Alamar blue”);
3. Method and instrument information;
  - A subgroup “size distribution”, providing placeholders for size distribution measured for the sample (including details of the dispersion protocol and dispersion medium). These fields are (almost) constant across all templates;
  - A set of parameters describing the experiment, including cell lines, instrument, controls, time points, concentrations. These differ widely across different experiments.
4. Results group: several columns to specify measured outcomes, along with measurement uncertainty;
5. SOP (reference to the protocol).

A separate set of Excel templates have been developed over the last decade at the Institute of Occupational Medicine (IOM) (https://www.iom-world.org/) and used to gather data in several EU FP7 projects (NANOMMUNE, NanoTEST, ENPRA, MARINA, NanoSolutions, SUN). They are being further adapted for use in more recent H2020 projects (currently PATROLS, GRACIOUS, BIORIMA, RiskGONE and NanoInformaTIX). These templates (referred to as IOM–Nano–EHS data templates) were originally derived in alignment with earlier (from 2009) JRC–IHCP NanoHub database requirements (built upon the IUCLID model, initially 5.1). These templates were based on the original OECD HT, for a variety of bio assays, and tailored to provide simplified subsets of the complete HT, and provided to the experimentalists for data collection spreadsheet format. This delivers a user friendly and suitably flexible approach whereby end-users entering data could modify the templates for the particular needs of their experimental regimes where necessary, and in liaison with the project’s database administrator. They have successfully provided a practical format for users collecting the results of a variety of physicochemical, in-vitro, in-vivo and eco-toxicology assays for nanoEHS safety assessment. Their flexibility and user friendliness increase user acceptability and utility, but potentially require an additional effort later in the data curation process, due to the need to process more variations in file layout and format to enable upload to the database. Whilst being laid out physically very differently from ISA–Tab, in order to facilitate the collection of data in patterns better reflecting the experimental conditions and data outputs, the templates still inherently share the logical principles and essential metadata features of ISA–Tab, including information on the test method and relevant SOPs as mandatory requirements. To date, the IOM templates have generally collected results for one substance and one assay type in a single Excel spreadsheet file composed of several related worksheets. The first worksheet (“Test conditions”) is organised in the following groups:

1. Experimental test and end point details. These collect general information about the project, work package, test facility, scientist(s) conducting the tests, start and end dates, details of assay name, specific endpoint(s), outcome metric(s), and experimental protocol;
2. Test substance/NM. The name, CASRN, project ID of the substance, with a separate field for additional general material IDs (e.g., JRC representative materials) and the highest concentration used;
3. Information on replicates and controls; Timeline and dosage/treatment concentration. The latter span several cells, as many as required to cover the time points and concentrations used. The names (T1, T2, T3, etc., C1, C2, C3 etc.) are used in data sheets to report the experiment outcomes;
4. Other experimental parameters may be required by the assay being recorded, including key items from the experimental protocol.

There are then experimental results data worksheets. These may differ in detail for different experiments. Generally, the raw data represents the experimental plate layout, indexed by T* and C*, and includes all replicates. The “Test results” worksheet typically includes formulae aggregating measurements’ results across replicates, and is organised in “blocks” (see Section 2.4 for details). The “Test summary” sheet is used to represent derived metrics over several concentrations, e.g., IC50.

### 2.2. The eNanoMapper Data Model

**Figure 1:**
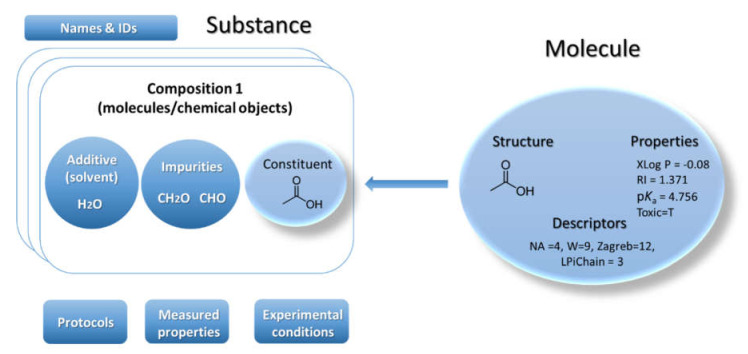
Substance data model/IUCLID inspired/(**left**) vs. Classical structure model (**right**).

The eNanoMapper database is an extension of the Ambit chemoinformatics platform [22]. Ambit can handle chemical structures via the classical model for representation of the molecule defined as a triad consisting of chemical structure, properties, and descriptors (Figure 1). Many chemical databases and QSAR models’ implementations are mostly based on this model. This classical paradigm has been used for several decades and it is still nowadays the predominant base layer for public chemical databases. However, the regulatory bodies require information on chemical substances as produced by the industry—not as a single chemical structure. Hence the classical paradigm for describing a molecule is not sufficient for a complete description of the substance. The substance paradigm is a more complex data model adopted in Ambit [23] to adequately represent the chemical objects under study. A more accurate representation of the composition and rich metadata is needed to effectively process data coming from many different biological analyses, including toxicity experiments. A substance is defined in the European Union regulation REACH [7] and in the Classification, Labelling and Packaging (CLP) Regulation as: “a chemical element and its compounds in the natural state or obtained by any manufacturing process, including any additive necessary to preserve its stability and any impurity deriving from the process used, but excluding any solvent which may be separated without affecting the stability of the substance or changing its composition”. According to REACH and CLP, a substance may contain one or more main constituents that make up a significant part of that substance. The main constituents are clearly distinguished from other types of constituents, which are impurities and additives. The REACH definition includes all forms of substances and materials on the market, including nanomaterials.

**Figure 2:**
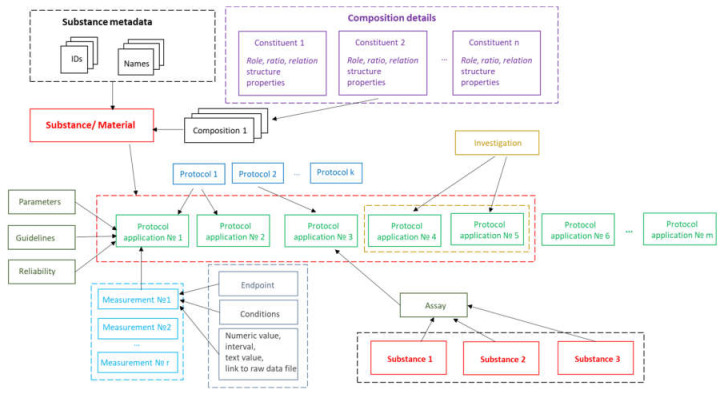
eNanoMapper/Ambit data model.

The implementation of substance support in Ambit is inspired by the OECD HT, CODATA, BioAssay ontology and ISA–Tab data model described above. The Ambit data model has been developed, optimized, tested, and improved for about a decade, processing many test cases and reflecting feedback from the users, all of which has helped to refine it. The eNanoMapper/Ambit data implementation has been designed to handle the major challenges encountered in the representation of chemical substances and nanomaterials. The eNanoMapper/Ambit data model is visualized in Figure 2. It is composed of a variety of data components (entities) serving different roles for the formalization, encapsulation, and serialization of items of information about particular substances/nanomaterials and measurements. The data model components (Figure 2) may have different representations at different stages of the information processing life-cycle: (1) JSON or RDF serialization on input or output; (2) Java classes in the server side Ambit implementation; (3) relational database entities (e.g., tables); (4) Python, R, Java, or JavaScript data structures when using the corresponding client libraries. Thus, the data model is a conceptual representation of chemical substances and measurements and can be implemented by different technology solutions, enabling data linking and interoperability. The Ambit cheminformatics platform uses these representations (1–3) internally and provides to external clients REST API representations in different formats (JSON, RDF, JSON–LD, ISA–JSON, TXT).

Substances are characterized by their composition and are identified by their names and IDs. The Ambit data model supports multiple names (e.g., public name, owner name) and multiple IDs as well as one or more compositions of the substance. The support for multiple compositions is in line with the IUCLIDv5 and v6 data model, complying with ECHA’s requirements. Each composition contains one or more components. Each component can be treated via the standard triad approach if needed (see Figure 1), i.e., specifying a well-defined chemical structure and properties. Complex relations between the substance components can be specified. Each component is assigned a role in the composition, such as main constituent, impurity, or additive, or, for cases of nanomaterials, the roles can be core, coating, functionalization, etc. Each component may have its own properties (typically properties calculated from chemical structure and identifiers), which are distinguished from the substance properties. The results from the physicochemical and biological measurements are treated as properties of the entire substance and are handled via the protocol applications.

The measurements data model has been described previously [8], but we highlight some of the essential points here for readers’ convenience. The event of applying a test or experimental protocol to a substance/nanomaterial is described by a “protocol application” entity (borrowed from the ISA–Tab model). Each protocol application consists of a set of “measurements” for a defined “endpoint” under given “conditions”. The measurement result can be a numeric value with or without uncertainty specified, an interval, a string value, or a link to a raw data file (e.g., a microscopy image). A particular “protocol application” is specified by a dynamic list of parameters as well as linking with Standard Operating Procedures (SOP), guidelines, publications, data quality flags, etc. The measurement conditions (or experiment factors, such as concentration, time, etc.) are another dynamic list of parameters, assigned to a different level of the data model. The data for particular NM may contain many “protocol applications”. The protocol applications that are related to one another can be grouped to form an “Investigation” entity. In addition, several different nanomaterials/substances that have the same “protocol application” applied can be grouped via the “Assay” entity (e.g., several materials and controls in the same assay). The high level components of the model, such as Substance, Protocol Application, Investigation, and Assay, have automatically generated UUIDs (hash based), which are used for linking and grouping the measurements.

**Figure 3:**
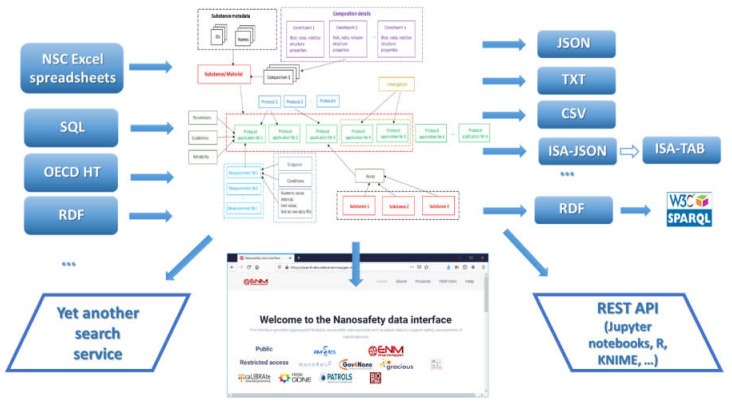
Data input and output to eNanoMapper/Ambit database.

The data model allows for integration of content from a variety of sources: OECD HTs (IUCLID5 and IUCLID6 files or direct retrieval from IUCLID servers), custom spreadsheet templates, SQL dumps from other databases, custom formats, provided by partners (e.g., the NanoWiki RDF dump [24]). Once the data is imported into the eNanoMapper database, a variety of options for export, data conversion, data retrieval, and data analysis are available (see Figure 3). The data model described is flexible and allows a variety of views of the data implemented via a Web GUI based on the jToxKit [25] JavaScript library, as well as many customized methods for accessing the data through a REST API via external tools like Jupyter notebooks and the KNIME analytics platform.

Taking into account the fact that the use of spreadsheet templates for data entry is the preferred approach by the majority of the EU NanoSafety Cluster projects, we developed a configurable spreadsheet parser that facilitates user friendly data preparation and database upload, as described in the next section.

### 2.3. Configurable Excel Spreadsheet Parser (NMDataParser)

The NMDataParser tool is a configurable Excel file parser, developed in Java on top of the Ambit data model and with extensive use of the Apache POI library [26]. It was designed and developed as an open source library to enable the import of nanomaterial characterization and safety data from the Excel spreadsheets provided by the nanosafety community. Within the two formats outlined in Section 2.1, there can potentially be unlimited permutations in layout, data, metadata, and terminology used in the files. The parser enables conversion of the data stored in the supported set of spreadsheet templates into the eNanoMapper/Ambit data model (implemented internally as Java classes and JSON, RDF, and ISA–JSON serializations). It accommodates different row-based, column-based, or mixed arrangements of the data found in the Excel spreadsheet. The parser configuration is defined in a separate JSON file (the JSON configuration syntax is described in Section 2.4), mapping the custom spreadsheet structure into the internal eNanoMapper data model storage components: “Substance”, “Protocol Application”, “Measurement”, “Parameters”, and “Conditions”.

**Figure 4:**
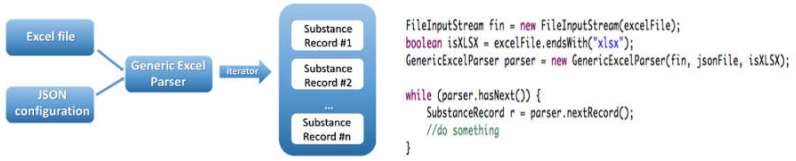
General usage of NMDataParser Java library.

The parser code, the JSON syntax, documentation and example files are available at https://github.com/enanomapper/nmdataparser/. This repository hosts Java code arranged as a multi-module Apache Maven project. The main package, *net.enanomapper.parser* (within the *enmexcelparser* module), contains the basic functionality of the parser. The parser can be started via the class GenericExcelParser, as shown in Figure 4. The parser works with a simple input: a spreadsheet (Excel) file and a JSON configuration file. As a result, an iterator to a list of substances (i.e., instances of the Java class SubstanceRecord from the Ambit Java data model) is returned.

The parser is embedded in the Ambit/eNanoMapper web application, allowing the upload of spreadsheets through a web form. The parser is also integrated into the main eNanoMapper import workflow (3.1).

The *enmconvertor* module is a command line application, which has options for converting input data in Excel, RDF, or JSON format into the eNanoMapper semantic model and serialization into eNanoMapper JSON, RDF, or ISA–JSON. Additional helper options are: extracting template fields of an input NanoReg/JRC template, generating a skeleton JSON configuration, and even generating an assay-specific Excel template. These helper functionalities are available as libraries in the *enmtemplategen* and *enmtemplate* modules.

The command line applications enabling file conversion without importing data into the database are useful for testing purposes, but also can be used to convert the input files into a common semantic model in a selected output format (JSON, RDF) with a subsequent storage and indexing outside of eNanoMapper database.

### 2.4. JSON Configurations for Data Import

The configuration metadata for the parser is defined in a separate file in JSON (JavaScript Object Notation [27]) format, mapping the custom spreadsheet structure into the internal eNanoMapper storage components. A comprehensive understanding of the data model is mandatory (see Section 2.1) for efficient use of the NMDataParser tool and to optimise the parsing process for successful database import. This section highlights the basic principles of NMDataParser configuration syntax based solely on the simplicity of the JSON format [27].

Additional resources with more sophisticated examples are given in the Supplementary Materials as well as the full documentation of the parser is available at https://github.com/enanomapper/nmdataparser/wiki.

The NMDataParser JSON configuration syntax includes a set of keywords (i.e., JSON attributes), specifying different strategies for reading the data from one or several Excel sheets, as well as allowing the combination of spreadsheet structures (sheets, rows, columns, blocks of cells, and individual cells) into the eNanoMapper data model. The JSON configuration file consists of several major sections, which are objects on the first level of the JSON schema:

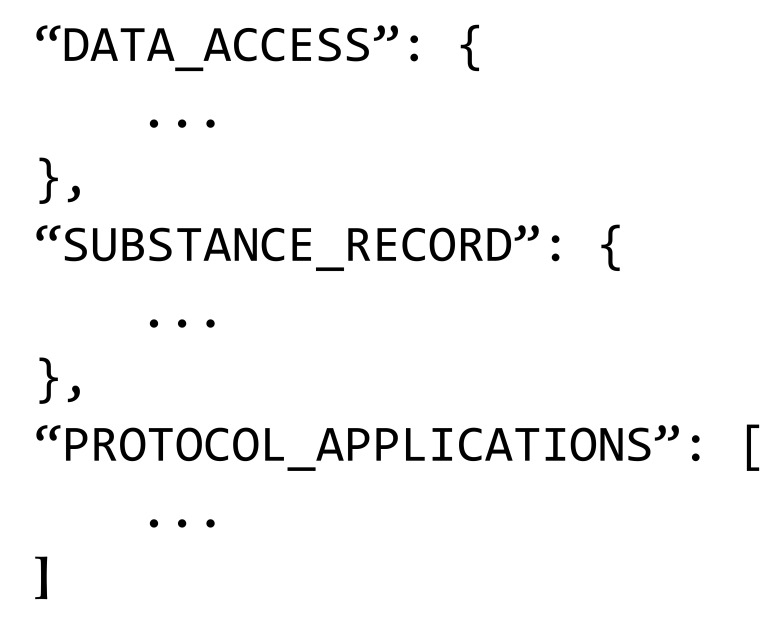



The section **DATA_ACCESS** defines the basic access to the spreadsheet template information, i.e., how data for the substance records (nanomaterials) is iterated. **SUBSTANCE_RECORD** is a section that defines data locations of the basic fields (components) of a substance record from the data model (see Section 2.1). The last major section on the first JSON level is an array of Protocol Application configurations stored as the JSON attribute **PROTOCOL_APPLICATIONS**. The Protocol Applications are associated with the Substance Record object, defined in the previous JSON section.

Most of the attributes in a JSON configuration are based on the concept of **Excel Data Location** (EDL). The EDL is a small JSON section (an object with several keys and values) that describes a particular location in the Excel workbook. EDL is similar to internal spreadsheet “coordinates”, specifying where and how to extract a particular piece of information, e.g., the endpoint within the Protocol Application entity is specified with an EDL as follows in the example:

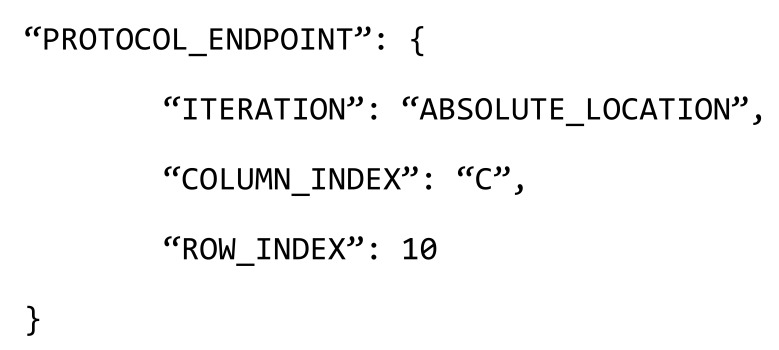



All major sections of the JSON parser configuration define attributes for particular components of the eNanoMapper/Ambit data model in the form:

<Data model entity (JSON attribute name)>: <Excel Data Location>

**Figure 5:**
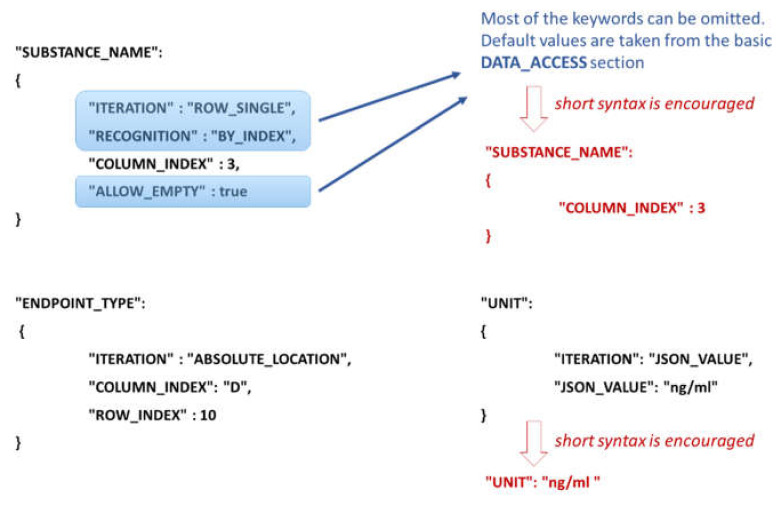
Excel Data Location definition for the attributes SUBSTANCE_NAME, ENDPOINT_TYPE and UNIT for three different iteration modes and short JSON syntax alternatives as well.

The NMDataParser JSON attribute names are identical to or closely resemble the eNanoMapper/Ambit data model components’ naming (i.e., names used in the SQL database schema or Java library classes; see also Figure 2). The EDL information depends on the iteration mode defined by the attribute ITERATION (Figure 5). A short syntax form is also allowed and encouraged for clearer, concise JSON configurations by omitting attributes that are defined globally by a DATA_ACCESS section (described below). For example, in the iteration mode ROW_SINGLE, only a column index is needed (actually, the row is not known in advance—it is obtained dynamically on the Excel rows iteration), while in the iteration mode ABSOLUTE_LOCATION, a column index and a row index are required. In this example, the Excel sheet index is not specified since it is taken globally (by default) from the DATA_ACCESS section as well. Figure 5 illustrates another commonly used iteration mode, called JSON_VALUE, i.e., the value for the data model entity (in this example UNIT) is given directly in the JSON configuration file. These are the most widely used modes of iteration. The EDL configuration syntax contains many more flexible options discussed further in the Supplementary Materials.

**The DATA_ACCESS** section defines the basic parameters for data access and iteration of the NM substances. The iteration mode is set through the attribute ITERATION and is used in every EDL object. NMDataParser supports several modes of iteration. Their implementation was motivated by the need to facilitate support for all known scenarios for accessing the NSC Excel sheets data (see more details in 2.1 Data entry templates and in the discussions in Section 3 and Section 4).

For most of the iteration modes (see Supplementary Materials Table S1), there is a primary sheet used to define the logic of reading (iterating) the substances from the Excel sheet. The DATA_ACCESS section describes attributes (keys) for the basic approach for reading Excel data and these are the default reading parameters (i.e., they may be omitted using short JSON syntax, as shown in Figure 5). When a particular attribute in EDL is not set, the default value is taken from the DATA_ACCESS section.

**Figure 6:**
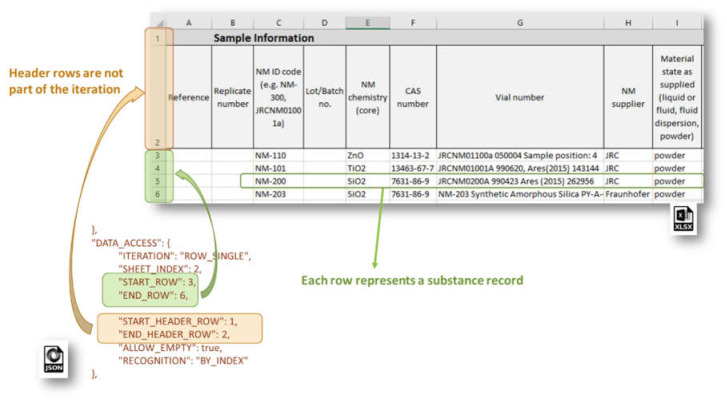
DATA_ACCESS configuration for iteration in the mode ROW_SINGLE.

The ROW_SINGLE mode is predominantly used for the NSC templates (Figure 6). The Excel sheet data is accessed treating each table row as a separate Substance record. The logic of data organization on a by-column basis of the substance data components is quite appealing and preferred in many cases (in Section 3 and Section 4 we discuss also the drawbacks of this approach). The header rows are not part of the iteration but the data therein can be accessed with an EDL in the iteration mode ABSOLUTE_LOCATION, for instance.

The NMDataParser tool recognizes missing or wrong types of attributes in the EDLs or other JSON sections and returns corresponding error messages. The error messages are crucial for the correct configuration of the NM data import and especially helpful when the JSON configuration is performed manually. The latter is quite common since domain expert knowledge is extensively used in parser configuration.

**Figure 7:**
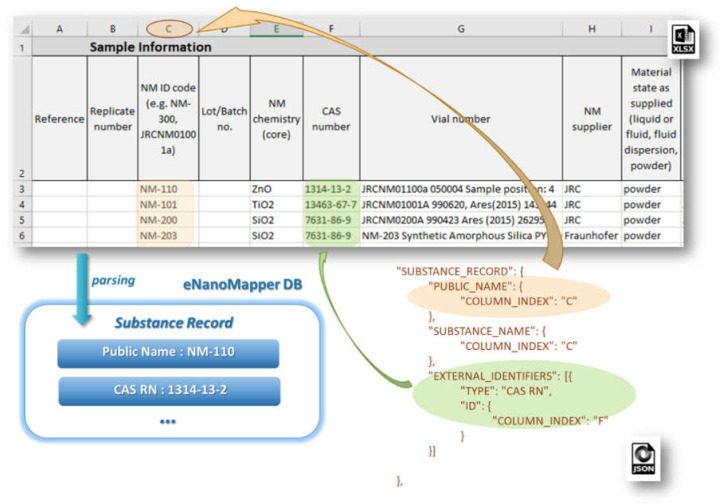
Mapping Excel data with EDLs in the iteration mode ROW_SINGLE and iterating Substance Records.

The **SUBSTANCE_RECORD** section contains a set of EDLs for reading the basic fields of a Substance Record, as described in the data model (see Figure 2). This section includes the attributes: SUBSTANCE_NAME, OWNER_NAME, SUBSTANCE_TYPE, OWNER_UUID, SUBSTANCE_UUID, PUBLIC_NAME, EXTERNAL_IDENTIFIERS, COMPOSITION. Figure 7 illustrates the configuration for the reading of NM name and CAS number mapped as an external identifier into the eNanoMapper database.

**Figure 8:**
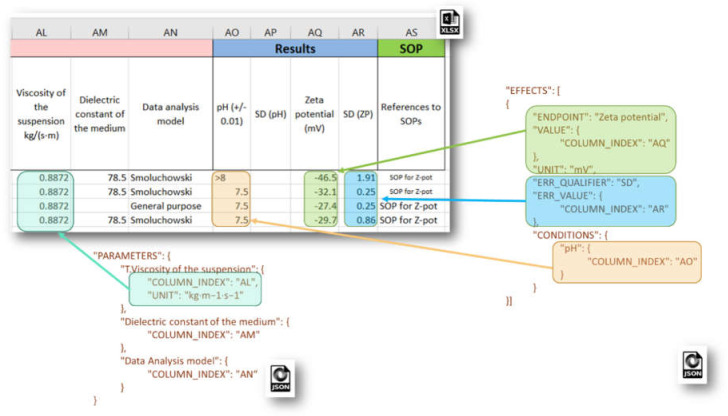
JSON configuration for Protocol Application parameters (**left**) and Effect Record (measurement) value, error value, and experimental conditions (**right**).

The **PROTOCOL_APPLICATIONS** section is an array of objects, defining all EDLs concerning Protocol Application data import. Each section includes attributes such as: CITATION_TITLE, CITATION_YEAR, CITATION_OWNER, INTERPRETATION_RESULT, INTERPRETATION_CRITERIA, PROTOCOL_GUIDELINE, PARAMETERS (an array of EDLs for the Protocol Application parameters—see an example from Figure 8), EFFECTS (an array of sections with EDLs for measurement data and associated metadata), etc.

Each **EFFECTS** section configures the mapping of spreadsheet data onto the Effect Record objects from the eNanoMapper data model, used for storing particular measurements, and includes EDL attributes such as: SAMPLE_ID, ENDPOINT, LO_VALUE, UP_VALUE, ERR_VALUE, TEXT_VALUE, VALUE, LO_QUALIFIER, UP_QUALIFIER, ERR_QUALIFIER, UNIT, CONDITIONS (an array of data locations for the experimental conditions). An example JSON configuration of experimental measurement of Zeta potential and corresponding metadata is given in Figure 8. The eNanoMapper Effect Record is a flexible data structure, which allows quite diverse measurement data storage. Each measurement may be a single number or an interval with lower and upper values, together with specified qualifiers, e.g., x = 3, x < 3, 3 < x ≤ 4 or 3 < x < 4, x~3, x = 3 + −0.5 etc. Measurement errors can be stored also with a separate qualifier, thus different approaches for setting the measurement uncertainty are supported (e.g., SD for standard deviation).

**Figure 9:**
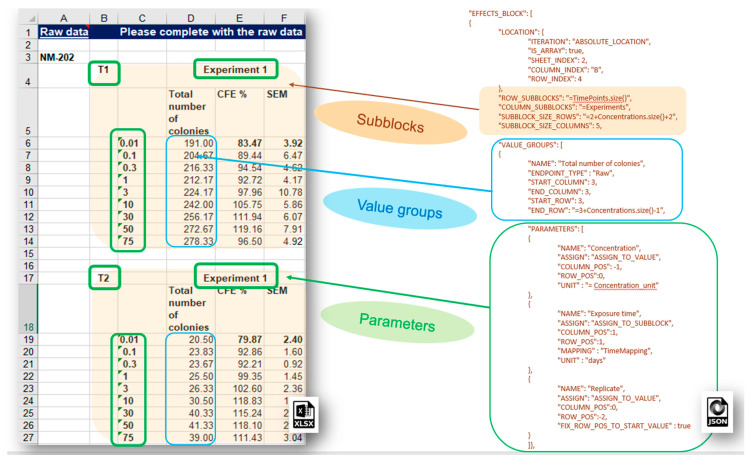
JSON configuration on an EFFECT_BLOCK; definition of sub-blocks, value groups and parameters of the value groups, which are the experimental conditions.

More complex data layouts of experimental measurements stored in spreadsheet files provided by the nanosafety community are often encountered. The typical bioassay experiment includes measurements with a variation of several experimental factors (the latter yields combinations of experimental conditions and multiple measurements), as in the dose response data recorded in the IOM–Nano–EHS Excel templates illustrated in Figure 9. The CFE (Colony Forming Efficiency) assay is performed using 2 replicates, 2 different timings, and 9 concentrations of silicon dioxide (i.e., 2 × 2 × 9 = 36 measurements to be reported for the full experiment). With some effort, it is possible to handle this layout using the EFFECT section described above, but it would be very inefficient and prone to human errors. The NMDataParser functionality was extended to support blocks of effects to avoid enumerating each combination of experimental conditions’ values by hand. A new JSON section EFFECT_BLOCKS is introduced within PROTOCOL_APPLICATIONS objects. It is used to configure a simultaneous reading of many effects, grouped in blocks of measurements according to the variations of the experimental factors. In this way, one EFFECT_BLOCK can describe multiple values found on one sheet. This feature is crucial for the configuration of HTS (high-throughput screening) data import where it would be practically impossible to handle each single value by hand.

The NMDataParser tool supports several levels of block data aggregation, which allows very complex spreadsheet data organizations to be mapped onto the eNanoMapper data model. Each block can be divided into a rectangular grid of sub-blocks. Each sub-block can contain several value groups. Each value group is a set of measurements where each measurement is associated with a list of experimental conditions called in this context parameters of the value group (as distinct from the parameters of the Protocol Applications).

Extensive description of the EFFECT_BLOCK usage and more examples are given in the Supplementary Materials.

## 3. Results

The *NMDataParser* tool is essentially enabling the most important stage of a FAIRification process [6]—mapping non-FAIR data (e.g., an Excel file) into a semantic model. A generic FAIRification workflow includes several steps: gaining access to the data, analysis of the data and metadata, defining a semantic model for the data and metadata, making data and metadata linkable, and deployment (hosting the FAIR data and providing human and machine readable access). We have developed a workflow encompassing these steps and have successfully applied it to integrate physicochemical, toxicity, exposure data and omics metadata from diverse Excel files provided by multiple completed and ongoing projects since the end of the FP7 eNanoMapper project in 2017. Access to the data is typically through bilateral project collaboration.

The analysis of data and metadata is an iterative process, requiring consultations with domain experts to explain the file content and layout, specifics of the assay, providing links to protocols and SOPs, and confirming correct ontology annotations of free text found in the files. The major advantage of the eNanoMapper/Ambit data model is a well-defined semantics, which have already been used to integrate large and diverse nanosafety data, with only minor enhancements/improvements related to annotation and arrangement of experiment entries introduced. In contrast, the initial *NMDataParser* [8] configuration files’ syntax was enriched with flexible options supporting various scenarios of the spreadsheet layouts. Hundreds of JSON configurations were defined manually or semi-automatically, stored in a version control system and included into the automatic import scripts’ unit tests. While one JSON configuration file can be applied to multiple Excel files with a similar layout, some complex spreadsheets (e.g., HTS) may require multiple JSON configurations for a single Excel file. The result of importing into a test database is verified for consistency through a set of automatic checkpoints, and, if necessary, the configurations are modified. This process is iterative, as confirmation on import correctness from data providers is usually required, and also the import configurations can be modified to address an issue reported by the database users. If an issue cannot be resolved, the configuration may include an option to set a quality flag. Where possible, the experiments are linked through the JSON configuration to SOP(s) that are available online. A modification in the JSON configuration results in an update of the release version for the particular dataset.

**Figure 10:**
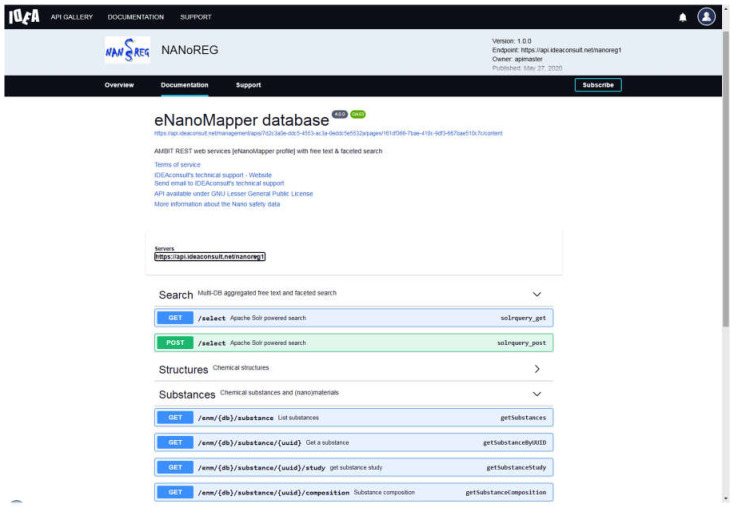
Interactive Application Programming Interface documentation at https://api.ideaconsult.net/.

Once the data is imported into an eNanoMapper database instance, it is immediately available (publicly or with a restricted access) via the web user interface and machine readable via an API supporting multiple serialization formats (e.g., JSON, RDF, JSON–LD). Interactive documentation of the API in OpenAPI v3 format is available at https://api.ideaconsult.net/ for each of the imported datasets (Figure 10). The access to datasets that are not intended to be publicly available is handled by an authentication and authorization system. The system supports both API keys and OAuth2 plans for either direct or delegated access grants.

### 3.1. Nanosafety Data Interface and eNanoMapper Database Instances

**Figure 11:**
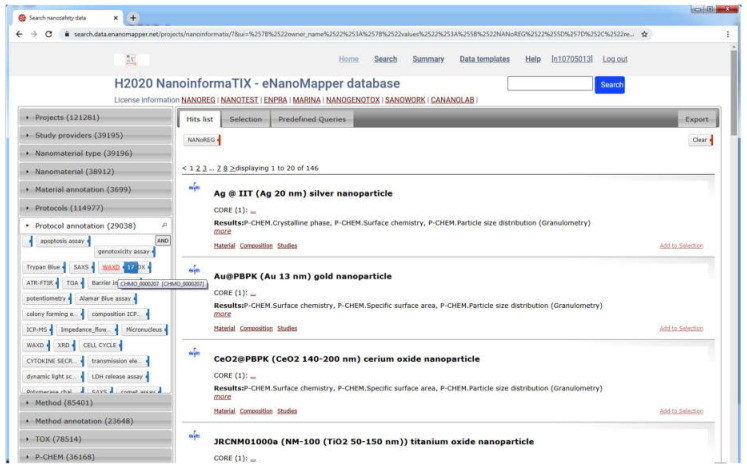
NanoInformaTIX–eNanoMapper database free text and faceted search interface, aggregating data from completed projects (NANoREG, ENPRA, MARINA, NanoGenotox, Sanowork) and US NIH caNanoLab. Ontology annotation of the WAXD is shown on the left. At the time of writing, the NanoInformaTIX database is only accessible to project partners.

The data from each project is imported into a separate eNanoMapper database instance intentionally; however, these instances serve as building blocks to feed the aggregated search of the overarching NanoSafety interface at https://search.data.enanomapper.net/, which provides a separate aggregated search API (Figure 10) and faceted search interface (Figure 11) based on Apache Solr [28]. Similarly, the RDF serialization can be used to feed a triple store and enable SPARQL queries.

**Figure 12:**
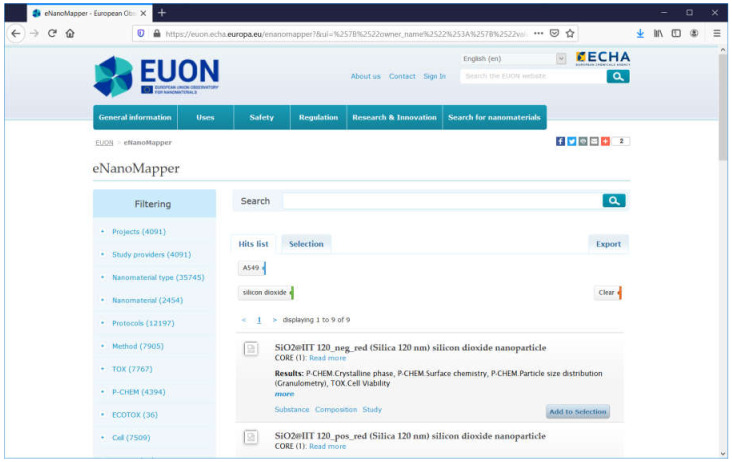
Screenshot of the EUON eNanoMapper database: result from a faceted search for records with silicon dioxide NM and experiments with cell lines A549.

A similar setup and faceted search interface was adopted by the EUON eNanoMapper database at https://euon.echa.europa.eu/enanomapper, which hosts publicly available content from NANoREG and eNanoMapper database instances (Figure 12).

**Figure 13:**
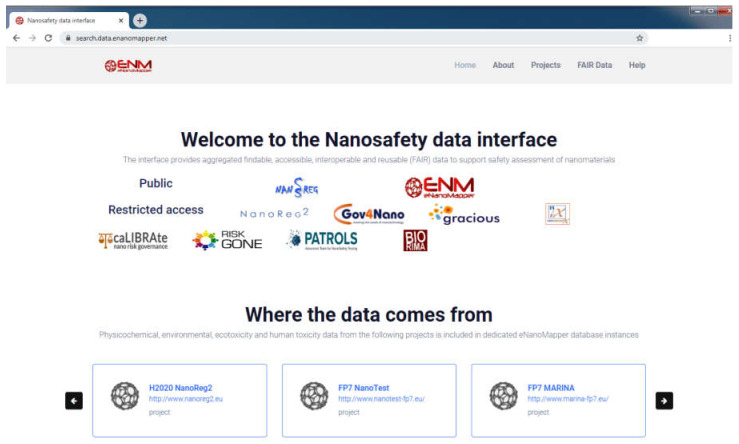
Screenshot of the entry page of the Nanosafety data interface to the eNanoMapper database’s web portal.

The nanosafety data interface available at https://search.data.enanomapper.net/ is an online user interface (Figure 13) enabling user friendly access to the aggregated search index of a (sub)set of eNanoMapper database instances (subject to access rights). Multiple project specific interfaces provide public (NANoREG, eNanoMapper) or protected access (NanoReg2, GRACIOUS, caLIBRAte, PATROLS, BIORIMA, RiskGONE, Gov4Nano, NanoInformaTIX—the latter presented in Figure 11).

Within the work of the NanoReg2 project, data from earlier completed FP7 projects NANoREG, NanoTest, MARINA, ENPRA, also from Nanogenotox, and the NanoReg2 data itself was curated and imported into eNanoMapper instances. The results of this work, including a description of the methodology, content, and relevant statistics, are described in a separate publication (submitted). The NanoReg2 manuscript includes an assessment of the FAIRness of the eNanoMapper database, with the conclusion that there is a high degree of compliance with the FAIR criteria. This result is consistent with the origin of the FAIR criteria in semantic technology and the focus on machine readability, which are inherent to the Ambit API design [22].

The SANOWORK project data was integrated via the work of the NanoInformaTIX project. caLIBRAte project data was integrated through collaboration between the caLIBRAte project and Ideaconsult Ltd. There are currently ongoing or planned activities for the integration of data generated by several H2020 projects: GRACIOUS, RiskGone, Gov4Nano, BIORIMA, PATROLS, and NanoInformaTIX.

### 3.2. Metadata Harmonization

Apart from the NANoREG SQL dump [29], NanoWiki RDF [24], and the remote integration with US NIH caNanoLab [29], all other data sources are in the form of ~1500 Excel (.xls or xlsx) spreadsheets, roughly following either JRC ISA–Tab logic templates, the IOM–Nano–EHS data templates, or a custom layout. Not surprisingly, thousands of spreadsheets, generated by hundreds of different organisations in various projects over more than a decade, which allow the use of free text for row/column names and cell values, result in great variation and inconsistencies. There is a huge inconsistency in the terminology used for many types of entities, including the naming of materials, endpoint, methods, cells types, concentrations, and time points, let alone specific protocol parameters, even within the same project and project partner. Our workflow uses ontology lookups and mappings on several layers. First, some alignment is done through the JSON configuration files, e.g., to ensure that “cell type” goes into the same field regardless of the naming in the Excel file. Second, the different objects at the output of the NMDataParser tool are subject to “dictionary” lookup, which is mainly to ensure different spellings of the entity are harmonized (e.g., harmonizing cell type “BEAS 2B”, “Beas 2B”, “BEAS -2B”). Next, the aggregated index is created with the help of yet another mapping procedure, which assigns one or more ontology annotations to the materials and different measurement entries (e.g., endpoints, methods), which can then be accessed through the aggregated search API. Finally, the eNanoMapper ontology [30] entries are used for synonym expansion in the aggregated search interface, adding to the flexibility of the free text search.

Two design decisions need to be highlighted. On import, we deliberately perform only minimal harmonization (spell checking, spelling variation, letter case variation, and abbreviation alignment) in order to store the values as close as possible to the original data source. The ontology annotation (besides material types and high level endpoints) is applied when the data is already imported into an eNanoMapper database, thus retaining the original meaning for subsequent human inspection, making annotation modifications and error fixing more flexible and even allowing specific annotations for different project views (e.g., using non-public dictionaries and ontologies). Finally, it is possible to annotate a database entry (e.g., method, endpoint) with more than one dictionary or ontology term. This was introduced to allow use of multiple dictionaries (including private ones) and increase interoperability with external software tools.

### 3.3. Export Formats and Libraries for Data Access

**Figure 14:**
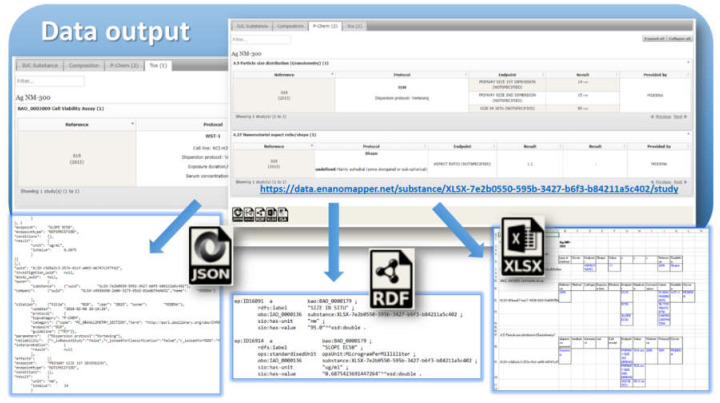
eNanoMapper database export: serialization as JSON, RDF, and Excel/CSV.

**Figure 15:**
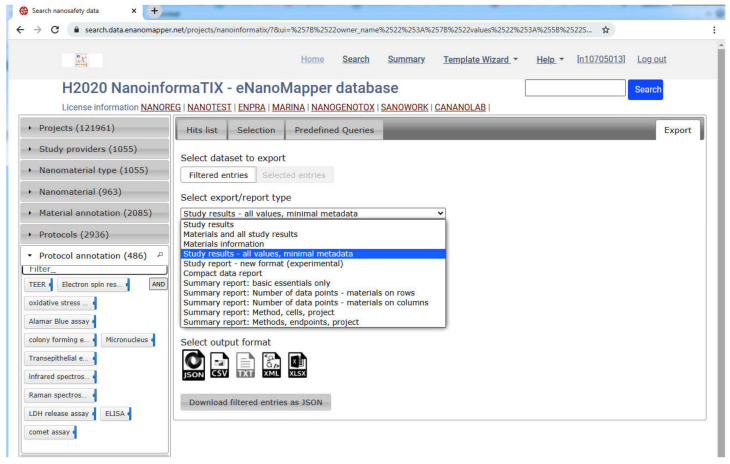
User friendly export facilities at the Nano Safety Data search interface.

Multiple data export formats are supported by the eNanoMapper database instances (both in the web user interface and the API), including the semantic formats (RDF, JSON–LD) and chemical structure and machine learning formats described previously [22], Excel file formats developed for the purpose of documentation of chemical substances read across workflow (Figure 14) [23], and JSON, the native serialization of the eNanoMapper/Ambit API (Figure 14 and Figure 15).

While manual editing of ISA–Tab files is tedious, the ability to export datasets in ISA–JSON (the JSON serialization of the ISA abstract model) enhances interoperability with external databases and data analysis workflows. ISA–JSON v1 export is supported by the Ambit API, using a nanomaterial extension schema developed by the authors [31], effectively the counterpart of the Material file in the ISA–Tab–Nano format. The nanomaterial extension schema describes all components of the nanomaterial; each component has a role (core, coating, etc.) and linkages to other constituents. The linkage describes the relation between two components. For example, two components may be covalently bonded, one may be embedded or encapsulated within another constituent, etc.

To facilitate data gap analysis and grouping, the aggregated search interface includes a number of options, allowing the exporting all of the search hits into JSON or spreadsheet formats (tab-delimited) as well as summary reports in Excel format. The definition of the summary reports depends on a flexible configuration and specific reports are being added upon request.

A number of open source libraries for accessing the eNanoMapper API are available, developed both during the eNanoMapper project—like the R (https://github.com/enanomapper/renm) and JavaScript bindings (https://github.com/enanomapper/ambit.js)—and after the project ended, like another JavaScript (https://github.com/ideaconsult/jToxKit) library and a new Python one (https://github.com/ideaconsult/pynanomapper). The Python library, in particular, is used for the set of open source Jupyter notebooks that demonstrate the eNanoMapper API (https://github.com/ideaconsult/notebooks-ambit/tree/master/enanomapper).

### 3.4. Availability

The Nanosafety search interface is available at https://search.data.enanomapper.net/. The eNanoMapper database instances and the Nanosafety search interface are hosted and maintained by Ideaconsult Ltd. The eNanoMapper database code is open source and available at http://ambit.sf.net. We provide Docker images and docker-compose configurations to allow convenient and quick set up of the required stack of software components. At present, the components included in the docker-compose setups are the Ambit platform and the eNanoMapper database, which also may be pre-populated with certain datasets, e.g., the freely available NANoREG data. More information is available in the GitHub repository: https://github.com/ideaconsult/ambit-docker.

## 4. Discussion

Data FAIRification is a complex process, requiring several areas of expertise—domain knowledge on the specifics of the data generation process: physicochemical characterization, eco and human toxicology, exposure, material science, as well as knowledge from the computer science domain—data modelling, software architecture, software services and their secure deployment, data protection and licenses. The need for multidisciplinary team involvement is obvious and, together with strong data stewardship skills, is recommended by several guides and publications [6,32]. However, this is a heavyweight process and data providers are frequently reluctant and slow to adopt workflows, tools, and data formats outside of their daily routine. The wet lab researchers initially generating results data rarely have sufficient knowledge of data modelling to appreciate the complexity of robust data entry tools and structured data formats. The focus on metadata, identifiers, and terminology lookup through ontologies in many FAIR guides is definitely useful and embraced enthusiastically, but the real bottleneck in FAIRification is the definition of the semantic model and conversion of the data files in hand to fit the data model.

The eNanoMapper/Ambit data model has been designed and implemented in order to accommodate the challenging features and attributes required to produce suitable chemical substance and NM databases, namely the handling of (i) Physicochemical identity (different analytic techniques, manufacturing conditions, batch effects, mixtures, impurities, size distributions, differences in the amount of surface modification, etc.); (ii) Biological identity (a wide variety of measurements, toxicity pathways, effects of ENM coronas, modes-of-action, interactions, cell lines, assays, etc.); (iii) Support processes requiring information (raw data, study summaries for regulatory purposes; linking with experimental protocols; risk assessment; grouping, safety-by-design) and (iv) support for data analysis (API and various types of views or interfaces such as “spreadsheet” or matrix, etc., merging multiple values, conditions, or similar experiments into a matrix).

The nanomaterials information workflow and data processing life-cycle poses many challenges due to the wide diversity encountered in its data sources, the many data input formats used, the wide range of data organization methods and the huge variety of modelling tools used on the data. We showcased two types of templates with different layouts (the JRC templates and the IOM templates with block organization of the data). The solution is an approach that enables **mappings** (i.e., eNano**Mapper**) between the different data formats and data presentations. To enable efficient mapping, a sufficiently flexible and generic data model is needed—one that can dynamically fit the requirements of many different external data formats. This is, namely, the eNanoMapper/Ambit data model. It is a flexible and general data structure that can describe measurements, with dynamic (not fixed) fields for endpoints, experimental conditions, protocol application, and several more metadata fields, annotated by ontology entries. The eNanoMapper data model is a generic description of any measurement and does not enumerate predefined fields for recording the results of a particular experiment. The latter (what are the essential parameters to describe an experiment ?) is a domain specific scientific question: how to represent the selected “aspects of the reality”.

Efficient aggregation of NM data from multiple sources (as is the case in data generated from past and ongoing NSC projects) is possible only if the original measurements are coupled with rich metadata. Metadata management is at the core of the FAIR initiative, while the efforts of the experimentalist are primarily focused on experimental data generation. Metadata that is easily findable, accessible and interoperable makes reuse of the experimental data feasible in a more practical manner for solving serious scientific problems, data gap filling, read across applications, QSAR/QSPR modelling and in supplying tools for other needs of the science, industry and regulators in the context of Safe-by-design NMs. Rich metadata layers, which are easily understood on a conceptual level and to be used practically, are one of the major milestones of the FAIRification process. The experimentalists feel comfortable with their own data and metadata when working with Excel sheets, especially with known formats and templates, and in our experience are often intimidated by the complexity of the more sophisticated data models like ISA. We expect that this trend of extensive usage and preference towards Excel spreadsheets will prevail in the near future. However, this could be a stumbling block to the creation of FAIR data. NMDataParser is a tool that provides a healthy balance between the requirements of the scientists (to store their data on a regular basis with ease and assurance) and the crucial needs of the FAIRification process. The JSON configurable Excel parsing process is transparent to many of the players (in various stages) in the NM processing workflow but enables the database import of virtually any Excel spreadsheet.

Data import into an eNanoMapper database enables sophisticated data processing workflows based on computer science, logic, and data modelling, all informed by expert knowledge. eNanoMapper’s powerful tools for import and export are developed on top of the data model described (see Figure 3), allowing conversion between different formats and data models and, more importantly, enabling the integration of data from different NSC projects, which is urgently required to support NM data analysis for the purposes of Safe-by-Design. eNanoMapper’s flexible system also includes programmatic access (API) and a user friendly web interface.

The FAIRification workflow presented in this work is simplified in comparison to the GO–FAIR FAIRification process [6] and relies on a well-defined, tried and tested data model and open source implementation. In the users’ interest, we strived to retain the data entry formats as closely as possible to the wet lab practices, hearing and incorporating the users’ needs and alterations, moving the burden of mapping the semantic models from those researchers to the database stages of the data life cycle—and this is clearly enabled by the NMDataParser tool. While the entire data import workflow is automated once the configuration files and dictionaries are in place, the creation of these still involves manual work and is prone to errors. To eliminate these problems, we have developed a semi-automatic JSON configuration generator and are now working towards a fully automated harmonized Templates Wizard. The latter generates community approved spreadsheet templates, ready for data entry, together with ready-to-use JSON maps for their upload once populated, and featuresan integrated ontology lookup.

## 5. Conclusions

The FAIRification workflow was developed during the integration of large nanosafety data sets generated by completed and ongoing NanoSafety Cluster projects using a defined semantic data model (eNanoMapper/Ambit). The first lesson learned along this process as well as previous data integration activities is to distinguish between the wet lab expertise and the data modelling expertise. Defining a “semantic model” for a dataset is the critical third step of the GO FAIR FAIRification process, however “describing the entities and the relationships in the data accurately, unambiguously and in a computer-actionable way” requires data modelling expertise and at least some knowledge about the IT infrastructure and the tools that could be potentially applied. The state-of-the-art currently does recognise this expertise mismatch and tries to handle it through “data stewardship”, i.e., an external team or organisation takes the data from the data provider and transforms it into a structured machine readable format in accordance with a chosen or newly created semantic model. However, once such a semantic model is defined in a sufficiently generic manner, it is possible to automate the process and revert to the state where researchers handle their own data in a FAIR way. We consider the eNanoMapper/Ambit data model appropriate, as it was successfully used not only for nanosafety data integration, but managing chemical substances and safety data from ECHA dossiers. The presented workflow makes use of ontology annotation on several stages, either automatically or with users’ involvement. Harmonization of the terminology, controlled vocabularies, annotation with ontology terms and usage of unique identifiers are very important to address Findability, Interoperability and Reusability, but there could not be Interoperability and Reusability without either a common semantic model or the ability to automatically convert between semantic models.

The next lesson learned is to avoid imposing “structured” data entry formats, which are usually easy to parse, but are not native to the daily experience of wet lab researchers. Instead, we opted to create tools to facilitate the conversion of familiar spreadsheets into machine readable format, complying with a predefined data model. Community driven harmonization of the spreadsheets’ template content and layout for different types of experimental techniques is essential in this process. Adoption of the new Template Wizard developed by the authors is currently underway, in close collaboration with data generators and providers in several current projects and with a growing set of data entry templates. We propose to consider a data entry template a FAIR resource itself (findable, citable, with appropriate license and metadata, reusable and interoperable with the help of the parser).

The workflow features a multistage process of aligning data and metadata with domain specific ontologies and data formats. The data is human-accessible through a web user interface and machine readable through a REST API with authentication and authorization implemented using standard practices and protocols (API keys and OAuth2). The modular architecture with search integration enables project specific views of public and restricted content. Every new project can have an integrated view of past projects’ data through search integration, without the need to copy the data.

The eNanoMapper database and the NMDataParser tool are freely available, open source projects, and Docker images facilitate the installation of a database instance anywhere.

## Supplementary Materials

The following are available online at https://www.mdpi.com/2079-4991/10/10/1908/s1, JSON configuration for NMDataParser; Figure S1: Description of the attributes defining the grid of sub-blocks of the main effect block, Figure S2. VARIABLES and VARIABLE_MAPPINGS configuration (left) and usage in VALUE_GROUPS section (right) to get original “T1” and “T2” Excel content imported into 9 days and 11 days respectively; Figure S3. Setting column shifts for measurement error: value group of CFE% highlighted in blue, error values (SEM) highlighted in green; Figure S4. Relative addressing (assigning) of the parameters in Effect Block Value Group: top (green)-ASSIGN_TO_VALUE, middle (blue)–ASSIGN_TO_SUBBLOCK, bottom (orange)-ASSIGN_TO_VALUE of the first element of the value group (FIX_ROW_POS_TO_START_VALUE is true); Table S1: Supported ITERATION modes in NMDataParser, Table S2. Description of the attributes defining the grid of sub-blocks of the main effect block, Table S3. Major keywords (JSON attributes) used to define a VALUE_GROUP.

## Abbreviations

API	Application programming interface
CASRN	Chemical Abstracts Service Registry Number
CODATA	Committee on Data for Science and Technology
CSV	Comma-separated values
ECHA	European Chemicals Agency
EDL	Excel Data Location
EUON	European Union Observatory for Nanomaterials
FAIR	Findability, Accessibility, Interoperability, and Reusability
GUI	Graphical user interface
IHCP	Institute for Health and Consumer Protection
IOM	Institute of Occupational Medicine
ISA–JSON	Investigation/Study/Assay JavaScript Object Notation
ISA–Tab	Investigation/Study/Assay Tab-delimited
IUCLID	International Uniform Chemical Information Database
JRC	Joint Research Centre
JSON	JavaScript Object Notation
JSON–LD	JavaScript Object Notation for Linked Data
KNIME	Konstanz Information Miner
Nano–EHS	Nanotechnology Environmental Health and Safety
NIH	National Institutes of Health
NM	nanomaterial(s)
OECD	Organisation for Economic Co-operation and Development
QSAR	Quantitative structure–activity relationship
QSPR	Quantitative structure-property relationship
RDF	Resource Description Framework
REACH	Registration, Evaluation, Authorisation, and Restriction of Chemicals
REST	Representational state transfer
SOP	Standard operating procedure
SPARQL	SPARQL Protocol and RDF Query Language
UUID	Universally unique identifier
WAXD	Wide-angle X-ray scattering

## References

[1] Jeevanandam J., Barhoum A., Chan Y.S., Dufresne A., Danquah M.K. (2018). Review on nanoparticles and nanostructured materials: History, sources, toxicity and regulations. Beilstein J. Nanotechnol..

[2] ECHA Nanomaterials in Our Daily Lives. https://euon.echa.europa.eu/uses.

[3] Nanosafety at the OECD. https://www.oecd.org/chemicalsafety/nanosafety/47104296.pdf.

[4] Sudha P.N., Sangeetha K., Vijayalakshmi K., Barhoum A. (2018). Nanomaterials History, Classification, Unique Properties, Production and Market.

[5] Haase A., Klaessig F. (2017). EU US Roadmap Nanoinformatics 2030.

[6] FAIRification Process. https://www.go-fair.org/fair-principles/fairification-process/.

[7] What Is a Substance?—ECHA. https://echa.europa.eu/support/substance-identification/what-is-a-substance.

[8] Jeliazkova N., Chomenidis C., Doganis P., Fadeel B., Grafström R., Hardy B., Hastings J., Hegi M., Jeliazkov V., Kochev N. (2015). The eNanoMapper database for nanomaterial safety information. Beilstein J. Nanotechnol..

[9] Jeliazkova N., Jeliazkov V., Willighagen E., Smeets S., Munteanu C., Fadeel B., Grafström R., Kohonen P., Sarimveis H., Tsiliki G. The first eNanoMapper prototype: A substance database to support safe-by-design. Proceedings of the IEEE BIBM’14 2014 Workshop on Nanoinformatics for Environmental Health and Biomedicine 2014.

[10] European Chemicals Agency—ECHA. https://echa.europa.eu/.

[11] IUCLID Product. https://iuclid6.echa.europa.eu/bg/project-iuclid-6.

[12] OECD Harmonised Templates. http://www.oecd.org/ehs/templates/.

[13] Visser U., Abeyruwan S., Vempati U., Smith R.P., Lemmon V., Schürer S.C. (2011). BioAssay Ontology (BAO): A semantic description of bioassays and high-throughput screening results. BMC Bioinform..

[14] Abeyruwan S., Vempati U.D., Küçük-McGinty H., Visser U., Koleti A., Mir A., Sakurai K., Chung C., Bittker J.A., Clemons P.A. (2014). Evolving BioAssay Ontology (BAO): Modularization, integration and applications. J. Biomed. Semant..

[15] Rumble J., Freiman S., Teague C. (2015). Towards a Uniform Description System for Materials on the Nanoscale. Chem. Int..

[16] CODATA/VAMAS Joint Working Group on the Description of Nanomaterials. https://codata.org/initiatives/working-groups/nanomaterials/.

[17] Marchese Robinson R.L., Cronin M.T.D., Richarz A.-N., Rallo R. (2015). An ISA–Tab–Nano based data collection framework to support data-driven modelling of nanotoxicology. Beilstein J. Nanotechnol..

[18] Sansone S.A., Rocca-Serra P., Field D., Maguire E., Taylor C., Hofmann O., Fang H., Neumann S., Tong W., Amaral-Zettler L. (2012). Toward interoperable bioscience data. Nat. Genet..

[19] ISA–JSON Format. https://isa-specs.readthedocs.io/en/latest/isajson.html.

[20] Totaro S., Crutzen H., Riego-Sintes J. (2017). Data Logging Templates for the Environmental, Health and Safety Assessment of Nanomaterials.

[21] Gottardo S., Ceccone G., Freiberger H., Gibsin P., Kellermeier M., Guggiero E., Stolpe B., Wacker W., Rauscher H. (2019). GRACIOUS Data Logging Templates for the Environmental, Health and Safety Assessment of Nanomaterials.

[22] Jeliazkova N., Jeliazkov V. (2011). AMBIT RESTful web services: An implementation of the OpenTox application programming interface. J. Cheminform..

[23] Jeliazkova N., Koch V., Li Q., Jensch U., Reigl J.S., Kreiling R., Georgiev I., Hubesch B. (2016). Linking LRI AMBIT chemoinformatic system with the IUCLID substance database to support read-across of substance endpoint data and category formation. Toxicol. Lett..

[24] NanoWiki RDF. https://figshare.com/articles/NanoWiki_4/4141593.

[25] JToxKit. https://github.com/ideaconsult/jToxKit.

[26] Apache POI. https://poi.apache.org/.

[27] JSON (ECMA-404 The JSON Data Interchange Syntax). https://www.ecma-international.org/publications/files/ECMA-ST/ECMA-404.pdf.

[28] Apache Solr. https://lucene.apache.org/solr/.

[29] Morris S.A., Gaheen S., Lijowski M., Heiskanen M., Klemm J. (2015). Experiences in supporting the structured collection of cancer nanotechnology data using caNanoLab. Beilstein J. Nanotechnol..

[30] Hastings J., Jeliazkova N., Owen G., Tsiliki G., Munteanu C.R., Steinbeck C., Willighagen E. (2015). eNanoMapper: Harnessing ontologies to enable data integration for nanomaterial risk assessment. J. Biomed. Semant..

[31] Jeliazkova N., Kochev N. ISA–JSON v1 Nanomaterial Extension. https://github.com/ISA-tools/isa-api/tree/master/isatools/resources/schemas/isa_model_version_1_0_schemas/material.

[32] Jacobsen A., Kaliyaperumal R., da Silva Santos L.O.B., Mons B., Schultes E., Roos M., Thompson M. (2020). A Generic Workflow for the Data FAIRification Process. Data Intell..

