# Supplementary material for: Your Spreadsheets Can Be FAIR: A Tool and FAIRification Workflow for the eNanoMapper Database
Source: Nanomaterials (Basel). 2020 Sep 24;10(10):1908. doi: 10.3390/nano10101908 (PMC7601422; doi:10.3390/nano10101908)
Supplement: Supplementary file 1 [file nanomaterials-10-01908-s001.pdf]

nanomaterials

Supplementary Materials

Your Spreadsheets Can Be FAIR: A Tool and FAIRification Workflow for the eNanoMapper Database

Nikolay Kochev [1,2,*], Nina Jeliazkova [2,*], Vesselina Paskaleva [1], Gergana Tancheva [1], Luchesar Iliev [2], Peter Ritchie [3] and Vedrin Jeliazkov [2]

1 University of Plovdiv, Faculty of Chemistry, Department of Analytical Chemistry and Computer Chemistry, 24 Tsar Assen St, 4000 Plovdiv, Bulgaria; vessy@uni-plovdiv.net (V.P.); gerganatancheva1@gmail.com (G.T.)
2 Ideaconsult Ltd., 4 Angel Kanchev St, 1000 Sofia, Bulgaria; luchesar.iliev@gmail.com (L.I.), vedrin.jeliazkov@gmail.com (V.J.)
3 Institute of Occupational Medicine, Research Avenue North, Riccarton, Edinburgh EH14 4AP, UK; peter.ritchie@iom-world.org
* Correspondence: nick@uni-plovdiv.net (N.K.); jeliazkova.nina@gmail.com (N.J.)

JSON configuration for NMDataParser

(supplementary material to *section 2.4* of article *Your spreadsheets can be FAIR: a tool and FAIRification workflow for the eNanoMapper database*)

Full documentation of the JSON syntax for NMDataParser configuration is available at: https://github.com/enanomapper/nmdataparser/wiki.

The configuration for the parser is defined in a separate file in JSON (JavaScript Object Notation) format, mapping the custom spreadsheet structure into the internal eNanoMapper storage components. JSON is an open standard data interchange file format used to store and transmit data objects consisting of attribute–value pairs and array data types. Some of the main characteristics of the JSON format are: human-readable, lightweight, text-based, programming language independent syntax, very common data format with diverse range of applications, serves as a replacement for XML, and defines a small set of structuring rules for the portable representation of structured data.
The JSON configuration file consists of several major sections which are objects on the first level of the JSON schema:

```
"TEMPLATE_INFO": {

   ...

},
"DATA_ACCESS": {
   ...
},

"SUBSTANCE_RECORD": {
   ...
},

"PROTOCOL_APPLICATIONS": [
   ...
]
```

TEMPLATE_INFO is the first section and used for technical (administrative) purposes only. This section contains attributes including: NAME, VERSION, and TYPE.

Iteration modes

Excel data is read into Substance Records (i.e. nanomaterial records) using several modes of iteration.

Table S1. Supported ITERATION modes in NMDataParser.

Iteration mode	Description
ROW_SINGLE	Data is accessed assuming each Excel row is a separate Substance record
ROW_MULTI_FIXED	A fixed number of rows are treated as a separate Substance record
ROW_MULTI_DYNAMIC	A dynamic number of rows are used to load a Substance record (the number of rows may vary for each record)
ABSOLUTE_LOCATION	The data component is read from an absolute locationwithin the Excel file (sheet, row, and column must be defined)
JSON_VALUE	Data component is taken directly from the JSON configuration file
VARIABLE	Data component is taken directly from the list of variables loaded
SUBSTANCE_RECORD_MAP	Substance record list is defined via a mechanism using variables. This mode is used for mapping complicated effect blocks to a predefined lists of substances

Iteration modes ABSOLUTE_LOCATION, JSON_VALUE, and VARIABLE are not used globally in the DATA_ACCESS section, but are locally used in many EDLs for particular eNanoMapper data model components (see examples in Figure 5 from the article; in these cases ABSOLUTE_LOCATION and JSON_VALUE overwrite the globally set iteration mode ROW_SINGLE).

For example, in ROW_SINGLE mode, an EDL will require only column index, while in ABSOLUTE_LOCATION mode, all of the indices will be used: COLUMN_INDEX, ROW_INDEX, and SHEET_INDEX (see Figure 5 from the article). SHEET_INDEX can be omitted when a cell from the primary Excel sheet is accessed (as it is in Figure 5 from the article) while iteration in mode ABSOLUTE_LOCATION allows reading data from Excel sheets different that the primary one. Accessing data from sheets different to the primary sheet is necessary when the data is spread over several sheets by original template design (e.g. IOM-nanoEHS templates). The primary Excel sheet (set in the DATA_ACCESS section) defines the logic of iteration i.e. how the substances are recognized within the spreadsheet data organization.

Excel Data Location (EDL)

Explicitly set attributes in an EDL overwrite the default values given in the DATA_ACCESS section wherever such exception is needed. Most often, the default values of the fields ITERATION and SHEET_INDEX (when not supplied explicitly) are taken globally from the DATA_ACCESS section

The Excel data location may define an array of Excel cells. For this purpose, a Boolean JSON attribute, IS_ARRAY, is set to *true* (if missing, default is *false*). Accordingly, the fields ROW_INDICES and COLUMN_INDICES are used to define arrays of row and column indices. Since all row/column indices are given explicitly, consequent index numbers are not mandatory, hence a more complex logic for data gathering in the array can be applied. Column indices can be set in two ways: as integers (i.e. indices as numbers) or as column labels (as used in the Excel standard cell addressing). For example: "COLUMN_INDICES" : [2,3,4] or "COLUMN_INDICES" : ["B","C","D"] are both valid specifications. Also, for long consecutive ranges of indices an alternative, a more convenient, string based syntax is supported: "COLUMN_INDICES" : "2-40".

The EDL attribute SOURCE_COMBINATION may be set to *true* to define a piece of information obtained as a combination of data from different columns and rows. All data sources are concatenated into a single text value. If needed, the attribute COMBINATION_SEPARATOR may be used to set a separator string for source combination concatenation. The combination of data from several columns is particularly useful for defining UUIDs of various data model components.

JSON Section EFFECTS

The measurement values for the effects records also can be read as arbitrary text which allows more complex aggregated data to be imported as a string. NMDataParser supports an intelligent recognition of Excel cells content where data, units, intervals, errors, and qualifiers may be combined into a single cell or spread over separate Excel cells. For example, the string “< 30 nm” could be in a single cell, or “<30” and “nm” could be in two different cells, or “<”, 30, and “nm” could be in three separate cells. All the example cases are fully supported and such flexibility is crucial to take into account the variability in NSC NM experimental data reporting.

JSON Section EFFECTS BLOCK

JSON section EFFECT_BLOCKS is used to configure a simultaneous reading of many effects, grouped in blocks of measurements according to the variations of the experimental factors.
The EFFECTS_BLOCK is a single JSON object or an array. Each EFFECTS_BLOCK is defined by its LOCATION attribute configured as an EDL object, several attributes to define the sub-blocks grid (see table S2), and an array section VALUE_GROUPS. Figure 9 from the article and figure S1 illustrate how the main effect block is constructed of 2 x 2 sub-blocks (only 2 x 1 are shown in the figures; full example Excel files are available at https://github.com/enanomapper/nmdataparser/wiki). For correct configuration of the sub-blocks, the same number of rows and columns in each sub-block and the distances between them are needed (i.e. correct spreadsheet generation is mandatory).

Table S2. Description of the attributes defining the grid of sub-blocks of the main effect block.

JSON attribute	Description
ROW_SUBBLOCKS	number of individual sub-blocks in the main effect block in a row (line)
COLUMN_SUBBLOCKS	number of individual sub-blocks in the main effect block in a column
SUBBLOCK_SIZE_ROWS	number of rows contained in one sub-block
SUBBLOCK_SIZE_COLUMNS	number of columns contained in one sub-block

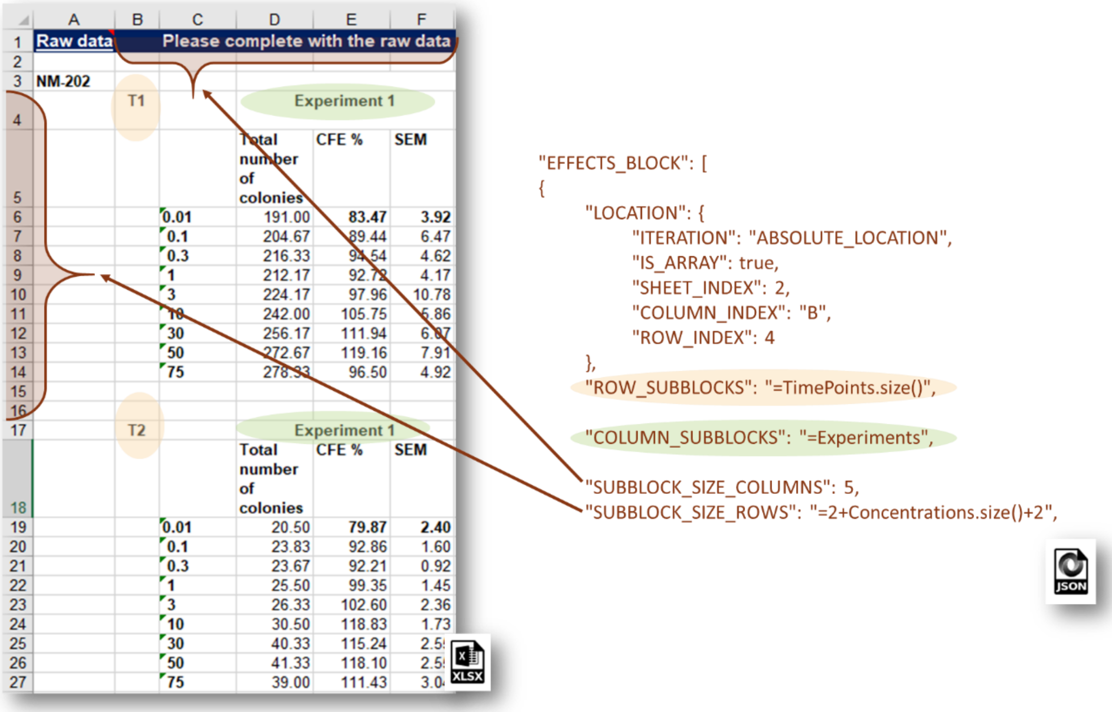

Figure S1. Sub-block definition in horizontal (columns) and vertical (rows) aspects.

The NMDataParser tool supports expressions with predefined variables to allow a more flexible definition of the blocks, sub-blocks and value groups. For example, in Figure S1, the SUBBLOCK_SIZE_ROWS attribute (the vertical size of each sub-block) is equal to 4 + number of concentrations used in the CFE assay. The following expression is used: **2 +** ***Concentrations.size()*** **+ 2** i.e. count 2 rows above and 2 rows beneath the main group of measurements spanning all concentration values: 0.01, 0.1, 0.3, …, 75 ug/cm^2. Hard coding the block and sub-block sizes is always an option (e.g. use directly 13 instead of *"=2 + Concentrations.size() + 2"*), but expressions usage gives more flexibility and allows reuse of the same JSON configuration for parsing multiple Excel files. For instance, CFE experiments with different sets of exposure times, replicates, and concentrations will be parsed with the same JSON if expressions are used.

Using variables

The VARIABLES key is an additional JSON array attribute within the section DATA_ACCESS which is a dynamic list of EDLs for all variables needed in the Excel reading/iteration process. The variables are used for constructing expressions in the Effect Block configuration (as shown in Figure S1). Also, the variables can be used for more complex routing of data e.g. the same value can be used in several components of the *Substance Record* but changed on a single place when JSON configuration updates are needed. Variable mappings (attribute VARIABLE_MAPPINGS) facilitate even more complex operations of the data import process. An example of variable mapping usage is given in Figure S2. It is quite common in the IOM-nanoEHS templates that the exposure times within effects blocks are designated as T1, T2, …, etc. while within the eNanoMapper database the real values should be imported. This is a typical case where variable mapping is applied in the JSON configuration.

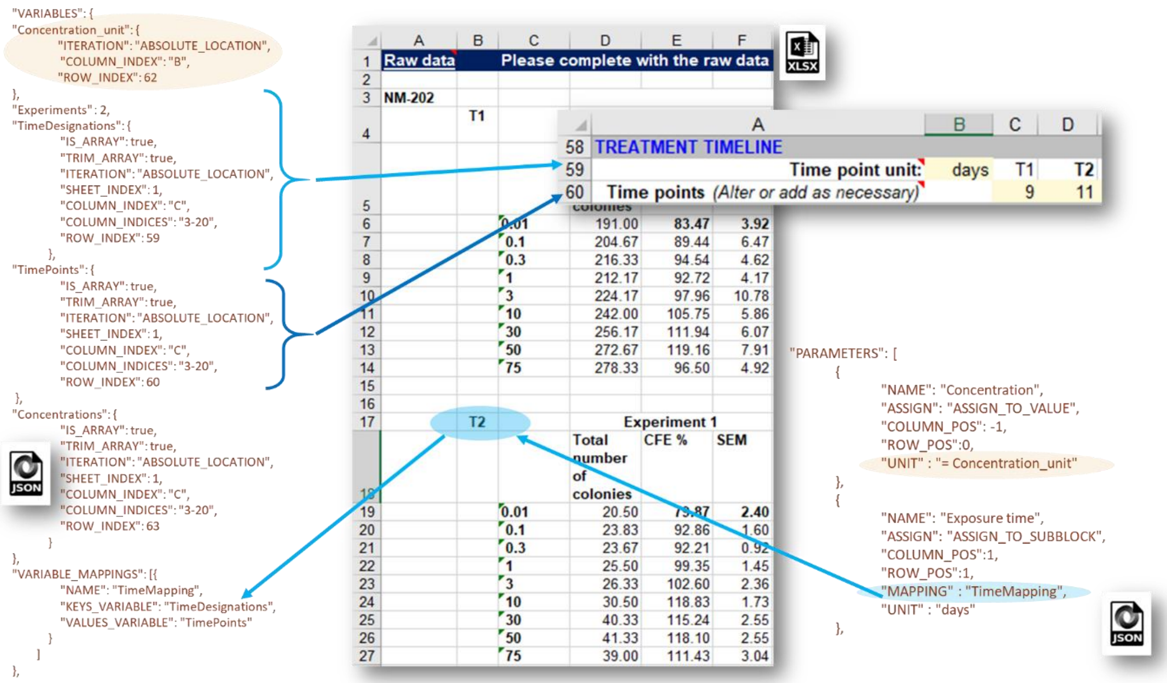

Figure S2. VARIABLES and VARIABLE_MAPPINGS configuration (left) and usage in VALUE_GROUPS section (right) to get original “T1” and “T2” Excel content imported into 9 days and 11 days respectively.

Value groups and experimental condition assigning

In a particular sub-block, each element of the VALUE_GROUPS array describes the experimental values for a separate endpoint measurement packed with a set of experimental conditions. Each value from the value group is imported into the database together with a separate combination of experimental conditions (Figure S3). Table S3 summarizes the fields needed for configuration of a value group. Most often the value group spans a single column (or single row) as shown in the examples in Figure S3 and Figure S4. However, it is possible to handle even more complex cases where a given value group spans a block of Excel cells containing more than one column/row and describes several different endpoints within the same Protocol Application.

Table S3. Major keywords (JSON attributes) used to define a VALUE_GROUP.

JSON attribute	Description
NAME	the name of the values (typically used to define the endpoint)
ENDPOINT_TYPE	the type of the endpoint (describes how the value is obtained, e.g. *average, row,* etc.)
UNIT	the measurement unit
START_COLUMN	the start column of the value group
END_COLUMN	the end column of the value group
START_ROW	the start row of the value group
END_ROW	the end row of the value group

The EDLs for value groups parameters (i.e. the experimental conditions) and some other effect blocks attributes use four different types of Excel data addressing called “assigning” defined be the attributes COLUMN_POS and ROW_POS. Basically the assigning defines what element of the effect block will be used as a starting point for the “coordinate system” used for relative addressing (i.e. defines the context for COLUMN_POS and ROW_POS) within the Excel sheet. The supported assigning types are:

- **ASSIGN_TO_EXCEL_SHEET –** parameter location is an address defined in the manner of basic EDL i.e. in terms of Excel sheets indices, column indices, and row indices;
- **ASSIGN_TO_VALUE** - parameter location is defined as relative shifts to the current individual value from the value group;
- **ASSIGN_TO_SUBBLOCK** - parameter location is defined as relative shifts to the beginning of the current sub-block.
- **ASSIGN_TO_BLOCK –** parameter location is defined as relative shifts to the beginning of the block.

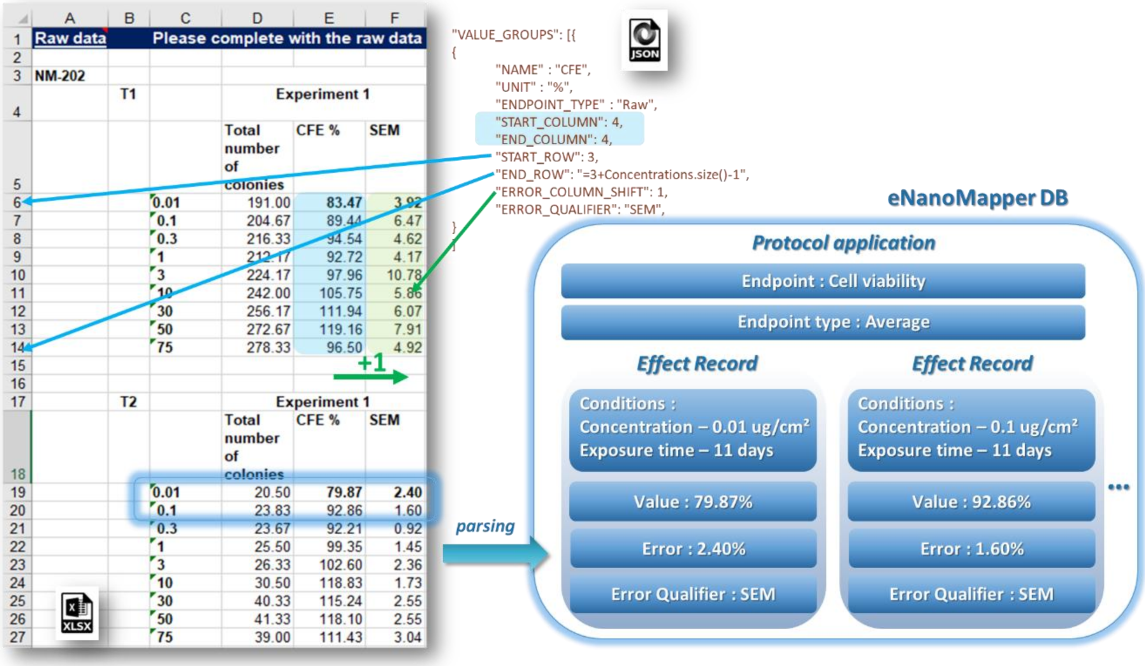

Figure S3. Setting column shifts for measurement error: value group of CFE% highlighted in blue, error values (SEM) highlighted in green.

Figure S3 illustrates the relative addressing of the position of the error values for a value group of measurements of the CFE assay. The value group is defined in the fourth column of the sub-block (START_COLUMN = END_COLUMN = 4) while the error values are placed in the column left to the values i.e. ERROR_COLUMN_SHIFT = 1 and ERROR_ROW_SHIFT = 0 (omitted in this case since it is the default value 0). The error shifts are always defined in assigning mode ASSIGN_TO_VALUE.

Figure S4 demonstrates variability of parameter assigning. Concentrations are spanned in parallel with the measurement values, hence ASSIGN_TO_VALUE mode is applied. Exposure time is the same for the entire sub-block, hence ASSIGN_TO_SUBBLOCK mode is used.

If needed, additional Boolean attributes FIX_ROW_POS_TO_START_VALUE and FIX_COLUMN_POS_TO_START_VALUE can be used to fix one of the dimensions (columns or rows) to the beginning cell of the value group as it is done for the "Replicate" condition from the example shown in Figure S4.

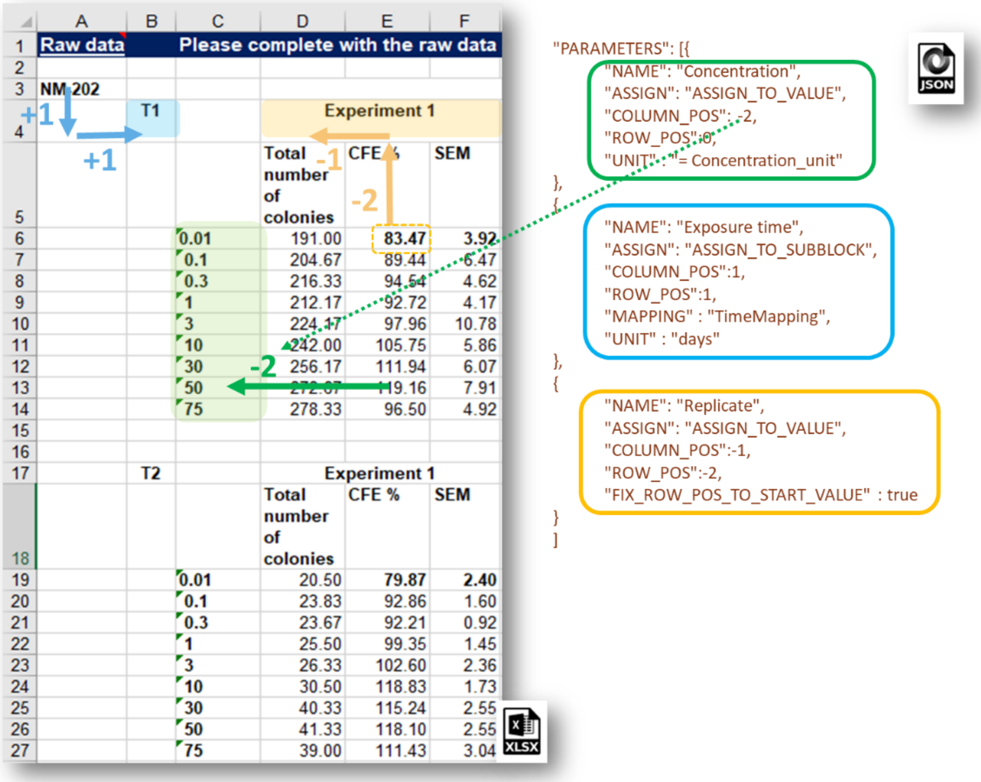

Figure S4. Relative addressing (assigning) of the parameters in Effect Block Value Group: top (green) - ASSIGN_TO_VALUE, middle (blue) – ASSIGN_TO_SUBBLOCK, bottom (orange) - ASSIGN_TO_VALUE of the first element of the value group (FIX_ROW_POS_TO_START_VALUE is *true*).
